# Deep Learning Spectroscopy: Neural Networks for Molecular Excitation Spectra

**DOI:** 10.1002/advs.201801367

**Published:** 2019-01-29

**Authors:** Kunal Ghosh, Annika Stuke, Milica Todorović, Peter Bjørn Jørgensen, Mikkel N. Schmidt, Aki Vehtari, Patrick Rinke

**Affiliations:** ^1^ Department of Computer Science Aalto University P.O. Box 15400 Aalto FI‐00076 Finland; ^2^ Department of Applied Physics Aalto University P.O. Box 11100 Aalto FI‐00076 Finland; ^3^ Department of Applied Mathematics and Computer Science Technical University of Denmark Richard Petersens Plads, 2800 Kgs. Lyngby Denmark; ^4^ Chair for Theoretical Chemistry and Catalysis Research Center Technische Universität München Lichtenbergstr. 4, D‐85747 Garching Germany

**Keywords:** artificial intelligence, DFT calculations, excitation spectra, neural networks, organic molecules

## Abstract

Deep learning methods for the prediction of molecular excitation spectra are presented. For the example of the electronic density of states of 132k organic molecules, three different neural network architectures: multilayer perceptron (MLP), convolutional neural network (CNN), and deep tensor neural network (DTNN) are trained and assessed. The inputs for the neural networks are the coordinates and charges of the constituent atoms of each molecule. Already, the MLP is able to learn spectra, but the root mean square error (RMSE) is still as high as 0.3 eV. The learning quality improves significantly for the CNN (RMSE = 0.23 eV) and reaches its best performance for the DTNN (RMSE = 0.19 eV). Both CNN and DTNN capture even small nuances in the spectral shape. In a showcase application of this method, the structures of 10k previously unseen organic molecules are scanned and instant spectra predictions are obtained to identify molecules for potential applications.

## 1 Introduction

Spectroscopy is central to the natural sciences and engineering as one of the primary methods to investigate the real world, study the laws of nature, discover new phenomena and characterize the properties of substances or materials. Spectroscopic materials properties must be known to design novel applications. For example, bandgaps are critical for solar cells, optical spectra for organic electronics, vibrational spectra to discover new thermoelectrics for waste heat recovery, X‐ray spectra for better medical diagnostic materials, or conductivity spectra for light‐weight batteries with high storage capacity.

Different spectroscopic techniques reveal different properties, and every material is characterized by a variety of spectra. Current spectroscopic methods, such as absorption, emission, scanning tunneling, Raman or electron‐paramagnetic resonance, are well established. However, experiments are often time‐consuming and sometimes require large, multi‐million‐Euro facilities, such as synchrotrons. Complementary theoretical spectroscopy methods based on quantum‐mechanical first principles are similarly time consuming and require large‐scale, high‐performance computing facilities.

Spectroscopy has seen many technical advances in individual spectroscopic methods, but no recent paradigm shift that would overcome the time‐cost conundrum. Here we show that artificial intelligence (AI) has the potential to trigger such a conceptual breakthrough toward data driven spectroscopy. We present the first step toward building an AI‐spectroscopist to harvest the wealth of already available spectroscopic data. The AI‐spectroscopist is based on custom made deep neural networks that learn spectra of organic molecules. Our neural networks predict the peak positions of molecular ionization spectra with an average error as low as 0.19 eV and the spectral weight to within 3%. This accuracy is already sufficient for our example application on photoemission spectra, which typically have an experimental resolution of several tenth of eV and theoretical error bars of 0.1–0.3 eV. Once trained, the AI‐spectroscopist can make predictions of spectra instantly and at no further cost to the end‐user.

In this new paradigm, deep learning spectroscopy would complement conventional theoretical and experimental spectroscopy to greatly accelerate the spectroscopic analysis of materials, make predictions for novel and hitherto uncharacterized materials, and discover entirely new molecules or materials. We demonstrate this by using our AI‐spectroscopist to make predictions for a new dataset of organic molecules that was not used in training the deep neural networks. At no further computational cost, we make spectra predictions for the 10 000 molecules of the diastereomers dataset of Ramakrishnan et al.^1^, ^2^ This gives us an overview over the spectral characteristics of the new dataset and helps us to identify interesting molecules for further analysis. In the future, we could extend this quick screening application to large numbers of organic molecules whose spectra have not been measured or computed, but are required for developing an application or analyzing an experiment.

## 2 Previous Machine Learning Attempts for Spectral Properties

AI methods, which encompass machine learning methods, are gaining traction in the natural sciences and in materials science.^3^, ^4^, ^5^, ^6^, ^7^, ^8^, ^9^, ^10^, ^11^, ^12^, ^13^, ^14^, ^15^, ^16^, ^17^, ^18^, ^19^, ^20^, ^21^, ^22^, ^23^, ^24^, ^25^, ^26^ However, previous work has focused on scalar quantities such as bandgaps and ionization potentials. For solids, only bandgap values and densities of states at the Fermi level have been learned with kernel ridge regression,^18^, ^26^, ^27^ support vector machines,^28^ reduced‐error pruning trees and rotation forests,^19^ gradient boosted decision trees,^25^ and Bayesian optimization.^23^ For molecules, kernel ridge regression^29^ and neural networks^5^, ^24^ have been applied to learn ionization potentials and electron affinities or nuclear magnetic resonance (NMR) chemical shifts.^30^ Both bandgaps and ionization potentials are single target values. The learning of continuous curves, such as spectra, is not frequently attempted.

In this study we compare the performance of three deep neural network architectures to evaluate the effect of model choice on the learning quality. We perform both training and testing on consistently computed (theoretical) spectral data to exclusively quantify AI performance and eliminate other discrepancies, unlike an early study^31^, ^32^ which compared predictions from theory‐trained neural networks against experimental data. In further contrast with early work,^31^ we probe model performance with dataset size by utilizing spectra for 10^5^–10^6^ organic molecules, sizes increasingly available from modern database resources.

## 3 Molecular Representation

In this work we approach molecules from an atomistic perspective, in which the atomic structure, that is coordinates of all the constituent atoms, is known precisely. This atomistic representation is natural to theoretical spectroscopy, as the spectral properties can then directly be calculated from approximations to the Hamiltonian of each molecule. In general, representation (or feature engineering) is an important aspect in machine learning. How to best present molecules and materials to an AI for optimal learning, prediction and inference has been a pressing question in chemistry and materials science for the last few years,^33^ and several different representations have been tried.^3^, ^4^, ^9^, ^21^, ^25^, ^27^, ^29^, ^31^, ^33^, ^34^, ^35^, ^36^, ^37^

**Figure 1:**
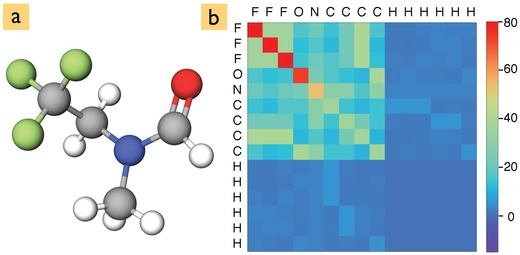
a) Atomic structure of the *N*‐methyl‐*N*‐(2,2,2‐trifluoroethyl)formamide molecule and b) its corresponding Coulomb matrix representation.

To represent the molecules to two of our three neural networks, we use the Coulomb matrix $C_{ij}=Z_{i}Z_{j}\|R_{i}-R_{j}{\|}^{-1}$, where *Z*_*i*_ is the atomic number (nuclear charge) of atom *i* and $R_{i}$ its position. The diagonal elements *i* = *j* have been fit to the total energies of atoms ($0.5Z_{i}^{2.4}$).^4^ A typical Coulomb matrix is shown in **Figure** **1** for the *N*‐methyl‐*N*‐(2,2,2‐trifluoroethyl)formamide molecule. The Coulomb matrix is appealing due to its simplicity and efficiency. We will show here that it provides sufficient input for learning molecular spectra, if the neural network architecture is sophisticated enough.

## 4 Method—Neural Network Architectures

**Figure 2:**
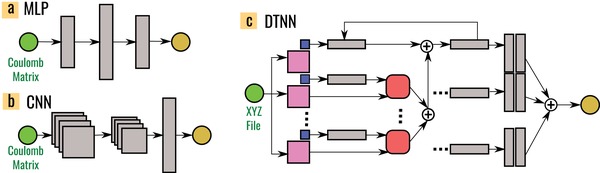
Canonical illustration of the three neural network types: a) the multilayer perceptron (MLP); b) the convolutional neural network (CNN); and c) the deep tensor neural network (DTNN). Green circles to the left represent the molecular input and yellow circles to the right the output (here 16 excitation energies or the molecular excitation spectrum). The gray blocks are schematics for fully connected hidden layers, convolutional blocks, pooling layers, and state vectors. Nodes corresponding to atom types in the DTNN are represented as blue squares and the distances matrix between different atoms as pink squares. Parameter tensors (red squares) project the vectors encoding atom types and the interatomic distance matrix into a vector with same dimensions as the atom type encodings. The DTNN is evaluated iteratively, building up more complex interactions between atoms with each iteration.

In this work, we chose neural networks due to their ability to learn complex mappings between input and target spaces (such as the Hamiltonian in quantum mechanics). Neural network models have surged in popularity recently, since they can express complex function mappings using inputs with very little or no feature engineering. Here we explored three neural network architectures illustrated in **Figure** **2**: a) the multilayer perceptron (MLP), which is one of the simplest architectures and accepts vectors as input, b) the convolutional neural network (CNN), which accepts tensors as inputs, and c) the deep tensor neural network (DTNN), a custom design for molecular data by Schütt et al.^22^ Each of the above is a deep network architecture. The depth, for example, in an MLP arises from stacking multiple hidden layers. Each hidden layer accepts output from the previous layer as input, and returns a nonlinear affine transformation as the output.

The MLP was chosen because of its architectural simplicity and also because a similar network was used earlier^5^ to predict fourteen different molecular properties simultaneously. Conversely, the CNN is the neural network of choice in image recognition. Much like an image, which is a matrix (or tensor) representation of a real world object, the Coulomb matrix is a matrix representation of a real molecule containing spatially repeating patterns, so we expect the CNN to perform well. The CNN transforms the input via a sequence of intermediate representations by convolving the input with one or more learnt filter matrices in a convolutional layer and passing the output through a nonlinear pooling operation in a pooling layer. Several convolutional and pooling layers are stacked and the final output is obtained by flattening the output of the last pooling layer via a fully connected layer (like the ones found in the MLP).

Making another conceptual leap, we adopt the DTNN architecture^22^ that has been motivated by previous architectures for text and speech recognition^38^, ^39^ and recently been used to predict atomization energies of molecules.^22^ In the DTNN, the atoms are embedded in each molecule like words in a text. The interaction between atoms and their surroundings are represented by an interaction tensor (the red block in Figure 2c) which is learned iteratively. Each atom in the molecule has its own interaction tensor, which in the first interaction pass encodes interatomic distances. In the second interaction pass the tensors learn angles between three different atoms and in subsequent passes higher order interatomic relations (e.g., dihedral angles). The DTNN encodes local atomic environments in a similar fashion as the many‐body tensor representation (MBTR) recently proposed by Huo and Rupp.^21^ However, the DTNN is designed to learn this representation rather than to expect it as input.

## 5 Training and Hyperparameter Optimization

The hyperparameters of each neural network (e.g., the number of hidden layers and nodes within them) are determined with Bayesian optimization for each dataset. This is a critical step, since it has been shown^40^ that effectively tuned network hyperparameters can achieve higher prediction accuracy than those with manually chosen ones. We used 90% of each data set for training and the rest was split equally between validation and test sets. The networks were trained by backpropagation, with the Adam^41^ update scheme. Root mean square errors (RMSE) and squared correlations *R* ^2^ were evaluated for the test set of molecules that the neural networks had not “seen” before. We take *R* ^2^ as a quality measure for the learning success of our neural networks, whereas the RMSE quantifies the predictive accuracy for excitation energies. We refer the reader to the Supporting Information for details on the DNN architecture, hyperparameters, and training algorithm.

## 6 Datasets

We use the QM7b^5^, ^42^ and QM9^2^, ^43^ datasets of organic molecules to train the AI‐spectroscopist. We optimized the structure of all molecules with the Perdew–Burke–Ernzerhof (PBE)^44^ density functional augmented with Tkatchenko–Scheffler van der Waals corrections (PBE+vdW)^45^ as implemented in the Fritz Haber Institute ab initio molecular simulations (FHI‐aims) code.^46^, ^47^ After discarding molecules with fewer than sixteen occupied energy levels, we were left with 5883 and 132531 molecules, which are henceforth referred to as 6k and 132k datasets, respectively. In each set we collect the highest 16 occupied PBE+vdW eigenvalues as excitation energies for each molecule. The molecular spectra are then computed by Gaussian broadening (0.5 eV) these eigenvalues into the occupied density of states. The resulting curve was discretized with 300 points between −30 and 0 eV. Level broadening encompasses vibrational effects, finite lifetimes and spectrometer resolution; we discuss our dataset choices in relation to our findings further on.

For the application test, we use the 10k diastereomers dataset of Ramakrishnan et al.^1^, ^2^ It contains 9868 “additional” diastereoisomers of 6095 parent C_7_H_10_O_2_ isomers from the 134k dataset.^2^ The molecules in this 10k set are not part of the 134k set and were used by Ramakrishnan et al. to validate their delta‐learning approach.^1^ We here use only the molecular coordinates from the 10k set and obtain the corresponding spectra with the trained deep learning framework.

## 7 Results

**Figure 3:**
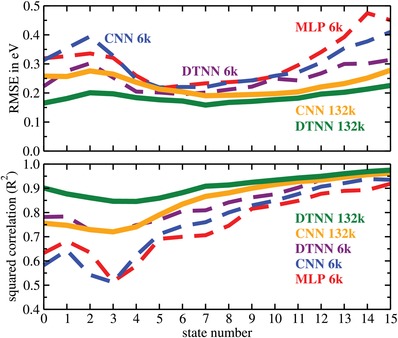
Root mean square error (RMSE) and squared correlation (*R* ^2^) for the sixteen molecular excitations for the different neural network architectures and data sets. The states are labeled in descending order from the highest occupied molecular orbital (state number 0).

First we discuss the simultaneous prediction of the 16 molecular eigenvalues in our datasets. **Figure** **3** shows the RMSE and *R* ^2^ values for the three different neural network architectures and the 6k and 132k datasets. We observe that only the DTNN 132k performs uniformly well across all 16 states. For the other networks the predictions of the deeper levels have the highest *R* ^2^ values and are therefore learned “best” regardless of the model and the dataset size. However, the predictive accuracy is still relatively low (high RMSE) for some networks. This seemingly contradictory behavior likely arises because lower energy levels (from 11 to 15) for smaller molecules correspond to electronic core states, which have a significantly higher absolute energy than valence states. While the cores states are easily learned, predictions with a low relative error at this end of the spectrum can result in absolute errors of several tens of eV and give rise to high RMSE values.

The learning quality then decreases gradually (the *R* ^2^ value decreases) the closer the state is to the highest occupied molecular orbital (state number 0) and then rises again from state 3 to 0. Interestingly, the RMSE exhibits an inverse correlation. It first improves and then rises again for the last 4 states. The best predictions are given by the DTNN 132k and have an RMSE of only 0.16 eV with an average RMSE of 0.186 eV (see **Table** **1**).

**Table 1:** Summary of the RMSE for the 16 excitations and the RSE for spectra for the 6k and the 132k datasets. The results are averages over 5 runs, except for the spectra predictions of 132k dataset which were averaged over 3 runs. The resulting statistical error is at most ±0.003 and has therefore been omitted from this table

Datasets →	6k		132k	
Model ↓	Levels [eV]	Spectra	Levels [eV]	Spectra
MLP	0.317	NA	NA	NA
CNN	0.304	0.057	0.231	0.039
DTNN	0.251	0.051	0.186	0.029

**Figure 4:**
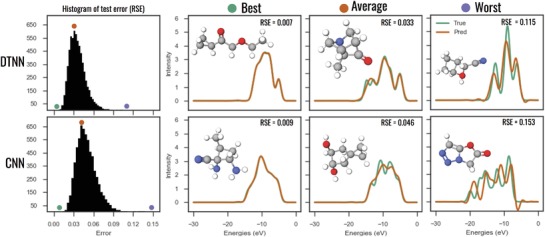
Comparison of CNN and DTNN spectra predictions: the first column depicts RSE histograms for 13 000 test molecules from the 132k dataset. The following three columns show the spectra of the best, an average, and one of the worst predictions compared to the corresponding reference spectrum. The colored circles mark the histogram positions of the selected molecules.

Next we consider spectra predictions for the CNN and DTNN trained on the 132k set as shown in **Figure** **4**. For spectra we calculate the relative difference (or relative spectral error (RSE)) between the predicted and the reference spectrum. The first column of Figure 4 shows RSE histograms for 13 000 test molecules from the 132k dataset. The RSE distribution is narrow and the typical error is around 4% for the CNN and 3% for the DTNN; very low for both neural networks.

To understand the spectra predictions better, we picked three spectra that are representative of the best, average and worst predictions made by the CNN and DTNN and plotted them in Figure 4 with the corresponding reference spectrum. We observe that the best predictions are able to capture all features of the reference spectrum. The average predictions for the CNN miss spectral features, but capture the average shape of the spectrum. The worst CNN predictions do not represent the reference spectrum well. The DTNN does much better in both categories. It captures most spectral features, but still averages through some.

Table 1 provides a performance summary for the neural networks we have tested. It confirms our observations that both the amount of training data and complexity level of the neural network improve the predictive power. The DTNN is our best performing network with an average error of 0.19 eV for energy levels and 3% for spectral intensity.

## 8 Application

To showcase the power of our deep spectra learning method, we present a first application of the AI‐spectroscopist. For the 10k dataset we have information on the structure of each molecule, but no spectra. Computing the spectra with DFT would take considerable computational effort and time. With the AI‐spectroscopist, we gain an immediate overview of the spectral content of the dataset.

**Figure 5:**
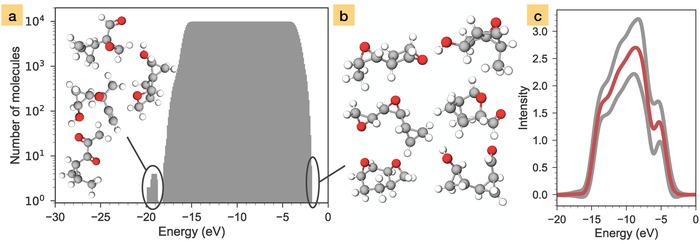
Spectral scan of the 10k diastereoisomer dataset performed with the DTNN: a) histogram of molecules that have spectral intensity at a certain energy. The four molecules in the inset are outliers that give rise to the peak with lowest energy. b) The six molecules that have the highest ionization energy. c) Average spectrum of all molecules in the dataset (red line). The gray lines mark the averages of the ±1 confidence level of the DTNN predictions.

A summary of the prediction is shown in **Figure** **5**. Panel a shows a histogram of the number of molecules that have spectral intensity (above a 0.1 threshold) at a given energy. It tells us that spectral intensity in this dataset is uniformly distributed between −18 and −2 eV for all molecules. Only four molecules have peaks below this range. The average spectrum, obtained by summing up all predicted spectra and dividing by the number of molecules, is shown in Figure 5c. This is the typical spectrum to expect from this dataset.

The spectral scan in Figure 5a also allows us to quickly detect molecules of interest in a large collection of compounds. The four molecules with spectral intensity below the main region and the molecules with the highest ionization energy can be easily identified, as illustrated in Figure 5b. Various molecules of interest, e.g., structures with peaks in particular regions of the spectrum, could then be further investigated with electronic structure methods or experiments to determine their functional properties.

In this fashion, the fast spectra prediction mode of our AI‐spectroscopist could be applied to the inverse mapping problem. Here, we seek to learn the structures of molecules or materials that exhibit certain properties. Inferring the atomic structure from a measured spectrum can be achieved with generative models,^48^ where AIs exposed to certain content are trained to produce similar content. However, most machine learning research to date has focused on generative models for continuous data like images and audio and not for the more difficult problem of generating discrete data such as molecules or materials. For solid clusters, simple inverse relations have recently been established between X‐ray absorption (XAS) spectroscopy^49^, ^50^, ^51^ and coordination shells of atoms. For molecules, neural‐network based auto encoders and decoders^52^ were combined with a grammar‐based variational autoencoder^53^ to map from the discrete molecular space into a continuous latent space (in which optimizations can be performed) and back. Even with such sophisticated models it is not easy to generate valid, synthesizeable molecules and inverse predictions remain difficult in practice.

The AI‐spectroscopist can facilitate inverse predictions for molecules, given a trial dataset of molecular structures with possible relevance. Spectral scan data can be produced at the press of a button, and analyzed for structures with the desired spectral features. These could then be screened for the best spectral match to produce candidate molecules. With scientific expertise and intuition, relevant trial datasets could be assembled for instant screening. Should it emerge that the trial dataset did not contain structure types associated with desired properties, the spectral scan search would require widening the structural pool. In such cases the search may not be successful, and it may be necessary to resort to a generative approach.

## 9 Discussion

Figure 3 shows a clear learning trend across the deep learning methods: with increasing NN sophistication (MLP→CNN→DTNN) the state dependence of both *R* ^2^ and RMSE reduces. Concomitantly, the learning success (increasing *R* ^2^) and the predictive accuracy increase (RMSE reduces). The DTNN is our most sophisticated network and trained for the larger 132k dataset it then exhibits the most uniform performance with high accuracy for every state.

Figure 3 also allows us to distinguish the effects of training set size and network complexity. Both the CNN and DTNN trained on the 132k dataset perform better than the corresponding models trained on the 6k dataset. As expected, their respective accuracies increase with the number of data points. However, the DTNN trained on only the 6k dataset almost outperforms the CNN trained on the 132k set. This illustrates that a purpose designed NN architecture can learn from fewer data points.

Regarding spectra predictions, even the worst predictions of the DTNN might still look good to a spectroscopist, as the overall shape and peak positions of the spectrum are captured well. The main differences between the DTNN prediction and the reference spectrum are slight peak shifts and an overall spectral weight reduction. Slight peak shifts lead to a large intensity difference, but only small difference in the peak energies, which is the more important observable in spectroscopy.

Our current spectral metric is very sensitive to peak positions. This is in principle desirable, since it forces the neural networks to prioritize on peak positions (and thus excitation energies). However, for many complex spectra, peaks due to individual excitations merge into a broader spectral structure. In such cases, it might be more suitable to adjust future metrics to better capture spectral shapes. In X‐ray diffraction (XRD) and low energy electron diffraction (LEED) studies the same problem arises, as theoretical spectra computed for model structures are compared to experimental spectra to find the best structural model. We will investigate the cosine or Pearson correlation coefficient and the Jensen–Shannon divergence measure^54^ as well as the Pendry R‐factor^55^ in the future. This will also help us to prevent negative peaks in the predicted spectral functions.

In this work we used the Kohn–Sham spectrum for simplicity. While Kohn–Sham eigenvalues do not correctly represent molecular excitation energies, they provide us with a convenient and large approximate dataset for developing and testing the AI‐spectroscopist. In the future, we will extend our study to photoemission spectra computed with the GW method.^56^, ^57^ Due to the much higher computational expense, we will always have more data from lower fidelity methods such as DFT‐PBE. To reconcile datasets at different fidelity levels, we are considering Δ‐learning techniques^10^ that would learn the difference between two different fidelity levels (here PBE and *GW* or co‐kriging techniques^23^, ^58^ that learn different fidelity levels simultaneously.

Our deep learning schemes are fully transferable to better accuracy computational datasets, but also to experimental spectra. We chose a relatively large broadening of computed electronic levels to mimic the resolution of common photoemission experiments, which produce broad and often fairly featureless molecular spectra. Future studies will address the effect of this broadening on the learning success, but our current findings indicate good quality predictions on broad spectral curves.

## 10 Conclusion

In summary, we demonstrated that deep neural networks can learn spectra to 97% accuracy and peak positions to within 0.19 eV. Our neural networks infer the spectra directly from the molecular structure and do not require auxiliary input. We also show that, contrary to popular belief, neural networks can indeed work well will smaller datasets, if the network architecture is sufficiently sophisticated. The predictions made by the neural networks are fast (a few milliseconds for a single molecule), which facilitates applications to large databases and high throughput screening. Our proof‐of‐principle work can now be extended to build more versatile AI‐spectroscopists.

## Conflict of Interest

The authors declare no conflict of interest.

## Supporting information

Supplementary
