# Supplementary material for: Deep Learning Spectroscopy: Neural Networks for Molecular Excitation Spectra
Source: Adv Sci (Weinh). 2019 Jan 29;6(9):1801367. doi: 10.1002/advs.201801367 (PMC6498126; doi:10.1002/advs.201801367)
Supplement: Supplementary file 1 — Supplementary [file ADVS-6-1801367-s001.pdf]

Copyright WILEY-VCH Verlag GmbH & Co. KGaA, 69469 Weinheim, Germany, 2019.

Supporting Information

for *Adv. Sci.,* DOI: 10.1002/advs.201801367

Deep Learning Spectroscopy: Neural Networks for Molecular Excitation Spectra

*Kunal Ghosh, Annika Stuke, Milica Todorović, Peter Bjørn Jørgensen, Mikkel N. Schmidt, Aki Vehtari, and Patrick Rinke**

Supplementary material
Deep learning spectroscopy : neural networks for molecular excitation spectra.

Kunal Ghosh,[1,2] Annika Stuke,[2] Milica Todorović,[2] Peter Bjørn Jørgensen,[3] Mikkel N. Schmidt,[3] Aki Vehtari,[1] and Patrick Rinke[2]

[1]*Department of Computer Science, Aalto University, P.O. Box 15400, Aalto FI-00076, Finland*
[2]*Department of Applied Physics, Aalto University, P.O. Box 11100, Aalto FI-00076, Finland*
[3]*Department of Applied Mathematics and Computer Science, Technical University of Denmark, Richard Petersens Plads, 2800 Kgs. Lyngby, Denmark*

(Dated: December 21, 2018)

In this supplementary material we describe the hyperparameters and training of the deep neural networks (DNNs). In general, we used 90 percent of the data for training and the rest was equally split between validation and test sets. The networks were trained by computing the gradients of the parameters with respect to the root mean squared error between DNN outputs and corresponding reference values. In the neural network literature, this procedure is usually referred to as backpropagation. The Adam [1] update scheme was used to update the parameters of each DNN. The initial learning rate for the Adam algorithm was also optimized and is listed in the corresponding sections below. We implemented our DNNs with the Lasagne [2] library, built on top of the Theano [3] deep learning framework. Unless otherwise stated, we use the default hyperparameter values from these libraries.

OPTIMIZED HYPERPARAMETERS

Each DNN has certain inherent hyperparameters such as the number of hidden layers and neurons within each layer. To obtain the best prediction accuracy, we optimized the hyperparameters of each DNN with Bayesian optimization (BO). This resulted in different hyperparameter combinations for each network (MLP, CNN and DTNN) and dataset size (6k , 132k). In the following sections, we list the hyperparameters used.

MLP

Our MLPs were build with the code from Ref. 4 and have two hidden layers and an output layer. Additionally, since the MLP accepts a fixed length vector as input, we binarized the randomized Coulomb matrices [5] to obtain such a fixed length vector for each molecule. The optimized MLP for **energy level prediction of the 6K dataset** had 250 units in each of the two hidden layers, and the sigmoid activation function was used in each of these layers. The best training mini-batch size was found (by BO) to be 30.

CNN

The CNNs have three convolutional layers followed by a max pooling layer. This combination is repeated three times. Then output of the last max pooling layer is passed into a fully-connected layer to generate the final output. The filters in the convolutional layers have a 3×3 size and a rectified linear unit or ReLU [6] activation function. Each max pooling layer has a pool-size of 2. The input Coulomb matrices were randomized following the scheme in appendix A of Ref. 7. Additionally, we set the maximum number of training epochs (one epoch is a complete pass through the training data) to 10,000 and stopped the training, if the validation error did not decrease for 100 epochs.

For **energy level prediction with the 6K dataset** the initial learning rate of the Adam algorithm and the training mini-batch size were found to be 1e-4 and 90, respectively. The optimum number of convolutional filters in each of the three consecutive convolutional layers were 22, 47 and 42, respectively. The same number of filters (22, 47, 42) were used in each of the subsequent sets of convolutional layers.

For **spectra prediction with the 6K dataset** the best initial learning rate (for Adam) and optimum mini-batch size were found to be 1e-5 and 105 samples, respectively. The optimum number of convolutional filters in each of the three consecutive convolutional layers were 37, 32 and 47, respectively.

The optimum hyperparameters for **energy level prediction with the 132K dataset** were 1e-2 and 90 for Adam's initial learning rate and training mini-batch size, respectively. The optimum number of convolutional filters were found to be 22, 47 and 42.

Finally, for **spectra prediction with the 132K dataset** we did not run the BO algorithm, because of very long training times. Instead we followed the settings for 132K energy level prediction. We set Adam optimizer's initial learning rate to 1e-4, training mini-batch size as 90 and convolutional filters in each of the three consecutive convolutional layers to 22, 47 and 42.

DTNN

Our DTNN implementation is similar to that of Ref. 8, but used three fully connected layers after the interaction passes to predict contributions from each atomic coefficient vector. Note that the number of units in the final hidden layer depends on the output dimensions, so we specify only the number of units in the first two hidden layers. In the first two hidden layers we used a hyperbolic tangent activation and in the final hidden layer a linear activation function. We set the number of interaction passes to two, i.e. encoded interactions up to angles. We also added noise to the distance matrix input to the DTNN during training. The noise was sampled from a normal distribution with zero mean and 0.1 standard deviation. The number of distance basis functions, over which the elements of the input distance matrix were expanded, was set to 40. The number of latent nodes in the tensor layer was 60. While training the network we set the maximum number of epochs to 10,000 and again stopped the training, if the validation error did not decrease for 100 epochs. The interested reader is refered to Section 3.3 of Ref. 9 for further details of the DTNN algorithm.

The optimum hyperparameter values for **energy level prediction with the 6K dataset** were as follows: The length of the atom coefficient vector (vector **c** in Ref. 8) was found to be 40, Adam's initial learning rate, training mini-batch size and the number of hidden units in the two hidden layers (described above) were found to be 1e-2, 190, 500 and 600 respectively.

As per the BO algorithm, **spectra prediction with the 6K dataset** was found to give the best results with an atom coefficient vector of length 37. With Adam optimizer's initial learning rate and training mini-batch sizes as 1e-3, 45 the number of neurons in the two hidden layers was 300 and 550.

For **energy level prediction with the 132K dataset** we obtained the best results with an atom coefficient vector of length 36 and 1e-4 as Adam's initial learning rate. The optimum mini-batch size and number of units in the two hidden layers were 50, 300 and 550 respectively.

Finally for **spectra prediction with the 132K dataset**, we did not run the BO algorithm, because of very long training times. Instead we followed the settings for 132K energy level prediction. We set the length of the atom coefficient vector to 40, the initial learning rate of Adam to 1e-5 and used a mini-batch size of 50 during training. The first two hidden layers used to predict contribution from each atomic coefficient vector had 100 and 200 units, respectively.

[1] D. P. Kingma and J. Ba, arXiv:1412.6980 [cs] (2014).

[2] S. Dieleman, J. Schlüter, C. Raffel, E. Olson, S. K. Sønderby, D. Nouri, *et al.*, "Lasagne: First release." (2015).

[3] Theano Development Team, "Theano: A Python framework for fast computation of mathematical expressions," (2016).

[4] `http://quantum-machine.org/code/nn-qm7.tar.gz` from website `http://quantum-machine.org/datasets/` Accessed : 26 April 2018.

[5] Binarization and Randomization of CMs was as per the scheme described in appendix A and B of [7].

[6] $\mathrm{ReLU}(x) = \max(0, x)$.

[7] G. Montavon, M. Rupp, V. Gobre, A. Vazquez-Mayagoitia, K. Hansen, A. Tkatchenko, K.-R. Müller, and O. A. von Lilienfeld, New J. Phys. **15**, 095003 (2013).

[8] K. T. Schütt, F. Arbabzadah, S. Chmiela, K. R. Müller, and A. Tkatchenko, Nat. Comm. **8**, 13890 (2017).

[9] K. Ghosh, *Deep Learning for Predicting Molecular Electronic Properties*, Masters Thesis (2017-10-23).
